# Validation of a Spatially Resolved Reflectance Imaging System for Recovery of *µ_a_* and *µ_s_*′ in Absorbing Turbid Media

**DOI:** 10.3390/s26072070

**Published:** 2026-03-26

**Authors:** Zachary D. Jones, Florian Foschum, Alwin Kienle

**Affiliations:** Institut für Lasertechnologien in der Medizin und Meßtechnik an der Universität Ulm (ILM), Helmholtzstr. 12, 89081 Ulm, Germany; zachary.jones@ilm-ulm.de (Z.D.J.); florian.foschum@ilm-ulm.de (F.F.)

**Keywords:** radiative transfer theory, scattering and absorption, diffuse reflectance

## Abstract

Many biomedical applications rely on the accurate recovery of absorption and scattering properties of human tissue. These characteristics serve as useful diagnostic indicators, holding information regarding the health and physiological status of a human subject. Many experimental methods exist for the determination of these optical properties, though many, such as integrating sphere methods, are not easily used in an in vivo setting. We have constructed and validated a spatially resolved reflectance imaging system that can be used to measure the absolute optical properties of absorbing turbid media in a non-contact, non-invasive fashion. We present detailed calibration procedures that consider our unique incident beam profile and system response with quantitative comparisons between experimentally and computationally obtained reflectance using Monte Carlo methods. Using highly scattering sphere suspensions with added absorption by ink, we show the spatially resolved reflectance imaging system’s ability to recover absorption within 20% of reference collimated transmission measurements and reduced scatter within 6% of those obtained by an extensively tested integrating sphere system, validating our system in preparation for in vivo measurements of the optical properties of human skin.

## 1. Introduction

### 1.1. Background

The radiative transfer equation (RTE) effectively describes light propagation in turbid media using the absorption coefficient *µ_a_*, scattering coefficient *µ_s_*, and scattering phase function, often approximated by the Henyey–Greenstein (HG) phase function, which is described by the single parameter *g*, referred to as the anisotropy factor [1,2]. Under such an approximation, the reduced scattering coefficient *µ_s_*′ can then be written as *µ_s_*′ = *µ_s_*(1 − *g*). Recovery of these parameters is essential for the optical characterization of turbid materials, and in addition to metrological applications, these optical properties also have great importance in biomedical applications. The absorbing and scattering characteristics of biological tissue hold information regarding their health and physiological status, allowing them to serve as useful diagnostic indicators [3,4,5,6,7,8,9]. The efficacy of optical transport models for biological tissues depends on accurately measured optical properties of in vivo human tissues, important for their application in improving biological sensing modalities, such as photoplethysmography and photoacoustic tomography [10,11,12,13,14]. Therefore, the accurate determination of in vivo optical properties of human skin, adipose tissue, and muscle is also important for the development and calibration of vital parameter sensing devices, such as pulse oximeters [15,16].

Many experimental methods exist for the determination of optical properties of scattering and absorptive media [17,18,19,20,21,22,23,24,25]. Most commonly, methods employ a technique described by one of the following: integrating spheres, goniometry, or diffuse reflectance sampling, whether in the spatial domain, time domain, spatial–frequency domain, or the temporal–frequency domain. Integrating spheres can provide the recovery of *µ_a_* and *µ_s_* by obtaining both reflectance and transmittance, integrated over all angles. Goniometry methods enable the measurement of angularly resolved reflectance and transmittance, allowing for the recovery of the complete scattering phase function, in addition to *µ_a_* and *µ_s_* [22]. Measurements of diffuse reflectance can recover the quotient of *µ_a_* and the reduced scattering coefficient *µ_s_*′, as only a single measured signal of reflectance is obtained.

The recovery of optical properties from measured light signals presents itself as an inverse problem, requiring one to solve the RTE. While analytical solutions for the RTE exist for some cases [26,27], for volumes of arbitrary optical and geometric complexity, numerical methods, such as Monte Carlo (MC), are required. In this case, an MC digital twin of an experimental system is constructed, and the difference between simulated and experimental signals is minimized, thereby solving the RTE to determine optical coefficients of an arbitrary sample. This difference can be minimized through an optimization algorithm, such as Levenberg–Marquardt (LM) or Particle–Swarm Optimization [28], by lookup table (LUT) matching from precalculated simulation data [29], or by neural networks (NNs) [30].

Each experimental method discussed above possesses unique advantages depending on factors such as measurement setting, time restraints, sample geometry, and system hardware, among others. Integrating sphere methods are thought to provide very accurate optical property recovery, but the hardware is rather bulky and sensitive, generally confining measurements to the laboratory. Goniometer systems provide the most detailed characterization of a scattering medium with a complete description of the scattering phase function, but system hardware is stationary, and measurement time is extended due to angular scanning and longer detector integration time at very large detection angles. In both methodologies, the sample is prepared with a thickness *d* such that the optical thickness *τ*(*λ*) = *d*∙*µ_t_*(*λ*) is neither excessively high, in order to ensure the presence of collimated transmittance so that *µ_s_* and *g* can be resolved, nor too low, such that diffuse reflectance and transmittance are sufficiently larger than the background. These restraints result in *d*, typically on the order of 0.1 to 2 mm, depending on the spectral range of measurement, for highly scattering materials, such as most biological tissues or tissue phantoms, e.g., intralipid dilutions [31]. In the context of biological tissue optical characterization, these restraints, along with the space restraints of the measurement system, result in such methodologies being ideal for excised tissues measured ex vivo, in which the sample may be cut to the required *d*. Diffuse reflectance methodologies, however, are the standard for in vivo optical characterization in which biological samples may be quite thick. As only the reflectance is collected, there is no upper limit of measurable *τ*, allowing utility for this technique as a method for probing the optical properties of materials for which a transmitted signal is not recoverable, such as a cross-section of human skin, including the deeper adipose and muscle layers.

Among the different measurement regimes of diffuse reflectance, this work focuses on spatially resolved reflectance (SRR) for the determination of optical properties. Often, SRR methodologies utilize a single light source supplied to a region of interest (ROI) and one or more detectors located at precisely measured source–detector distances (SDSs). Commonly, optical fiber bundles are used, in which a central fiber supplies incident light, and several fibers may be positioned at different SDSs. Imaging SRR systems have been constructed in the past to recover optical properties [23,32,33,34,35], giving the researcher access to a far greater number of distinct SDSs, dependent on the spatial resolution of a camera rather than the number of detection fibers. With light incident on a turbid sample, one can acquire the image of reflected light, which holds information regarding its optical properties. Imaging SRR methodologies provide flexibility in the range of samples that can be measured with the same hardware, as one can obtain the optical properties of samples of high and low attenuation by considering intensities at different pixels rather than manually adjusting hardware in the case of fiber-based approaches. This benefit comes at the expense of portability of the system, as a camera of high bit depth and low noise requires cooling, and the working distance (WD) must be precisely measured and kept constant across measurements. Considering the typically large WD (≥150 mm), however, commonly measured physiological ROIs, such as human forearms or hands, can easily be accommodated to enable the system to function well for in vivo optical characterization of biological tissue.

### 1.2. Motivations for This Work

Previous works have been published demonstrating the efficacy of SRR imaging systems for the determination of optical properties *µ_a_* and *µ_s_*′ [23,32,33]. These works have provided numerous elements of an SRR system that appear necessary for its functionality in the accurate determination of optical properties. Kienle et al. used a He-Ne laser emitting at 632 nm and a laser diode emitting at 751 nm as light sources, and a cooled CCD camera as the detector [23]. MC simulations were used to obtain reference reflectance, with optical coefficients determined using a pair of NNs. From this work, the importance of accurately measuring the incident beam profile enables absolute optical property fittings, eliminating the need to fit an additional scaling coefficient during each optimization. Pilz et al. demonstrated the importance of considering the point spread function (PSF) in optical coefficient fittings [33]. Here, using a 10 μm core diameter fiber focused onto a CCD sensor, the PSF of the camera system was measured, resulting in more accurate optical coefficients than when the PSF was not considered. Foschum et al. introduced a Xenon light source paired with a monochromator to allow a large spectral range of optical coefficient fittings [32]. This work provided an analysis of methods for measuring the PSF and introduced a flat-field correction for further improvement of signal quality, which was important to the accuracy of fitted optical properties.

In this work, we present and validate a new SRR system, which has been improved relative to earlier generations. In comparison to previous systems, an additional incident light monitoring photodiode was added to the system hardware to improve repeatability of measurements. Additionally, a camera with higher spatial resolution replaced that which was used in [32], improving the precision of measured reflectance for inverse fittings. We present measurements of the system response using two reflectance standards to compare their effect on fitted optical coefficients. Further, we integrate the measured beam profile directly into our MC model, by which we obtain simulated reference reflectance curves, allowing for the measurement of a so-called scaling coefficient that enables absolute fittings, thereby combining the utility of absolute fitting methodology of [23] while conserving the expanded spectral range and the rigorous treatment of optical system response of [32]. To validate the system, we performed optical coefficient fittings of highly scattering reference standards, with absorption ranging across multiple orders of magnitude. Our validation demonstrates the improvement of the system compared to previous generations in preparation for measurements of more sophisticated media, such as layered volumes and in vivo biological tissues.

## 2. Materials and Methods

### 2.1. Sample Preparation and Reference Measurements

For the validation of our SRR experimental system, we used sphere-suspension reference samples constructed with varying concentrations of a styrene acrylic hollow-sphere polymer suspension (Orgal Orgawhite 2000, MPA 881, Organik Kimya, Istanbul, Turkey) and an ink absorber (Brilliant black 4001, Pelikan, Berlin, Germany). In total, eight samples were made, with a naming convention according to their scatterer and absorber concentrations, as shown in Table 1. A more extensive commentary regarding the choice of reference validation sample can be found in the Discussion Section of this work.

An integrating sphere system was used to obtain reference values of the optical coefficients *µ_a_* and *µ_s_*′ for comparison with those measured using the SRR system. This system has been applied extensively, with a variety of samples [36], and extensive details regarding its methodologies can be found in recent published works [24]. Briefly, the system consisted of a proprietary 3D printed sphere with barium sulfate coating and three illumination ports for transmission, reflection, and normalization, and one detection port. A halogen lamp (Halostar Starlite, OSRAM, Munich, Germany) provided white light illumination, and scattered light was detected by a pair of spectrometers (Maya2000Pro & NIRQuest512-1.7, Ocean Optics, Orlando, FL, USA). The experimental system was paired with a custom digital twin MC code to obtain simulated reflectance. Optical properties were recovered from unknown samples using the minimization of a cost function via the LM algorithm. Prior to measurement, the stock solution from Table 1 was placed into an ultrasonic bath for 15 min to separate any agglomerated spheres and subsequently mixed in an industrial mixer (Hauschild SpeedMixer, Hamm, Germany) for 12 min to ensure a suspension with homogenously distributed scatterers. An integrating sphere sample was constructed by filling an aluminum spacer of a specified thickness *d*_IS_, sandwiched between two JGS1 glass slides, cleaned prior to the sample construction with soap and water, of 25.4 mm and 1 mm nominal diameter and thickness. Depending on the sample’s attenuation, *d*_IS_ ranged from about 0.5 to 2 mm. Care was taken to prevent the formation of air bubbles in the sample, and compressed air was used to remove dust from the glass surface prior to measurement. For each sample in Table 1, four measurements were taken and each input into an LM minimization algorithm to determine an average and standard deviation of fitted *µ_a_* and *µ_s_*′ over the four trials. For all samples, we used an anisotropy *g* = 0.8, applying the Henyey–Greenstein phase function and the refractive index *n*(*λ*) of water [37]. These values and their error bars were then reserved for reference values in this work.

In addition to the integrating sphere system, a collimated transmission system [38] was employed to obtain additional reference values of the absorption coefficient. Three samples were made by adding the nominal concentrations of the ink absorber listed in Table 1 to pure water and omitting the scatterer. As the ink is a molecular absorber, these samples exhibited no scattering behavior, allowing the measured extinction coefficient to be taken as the absorption coefficient. Each sample was constructed in a similar fashion to the integrating sphere samples, with 3D-printed plastic spacers of specified thicknesses *d*_CT_ between glass slides. In this case, *d*_CT_ varied from about 10 to 20 mm, depending on a given sample’s absorption. We note that the absorption of these samples may differ from that of the sphere suspensions due to the added ink quantities differing slightly from their nominal values.

### 2.2. SRR Imaging System: Hardware

**Figure 1:**
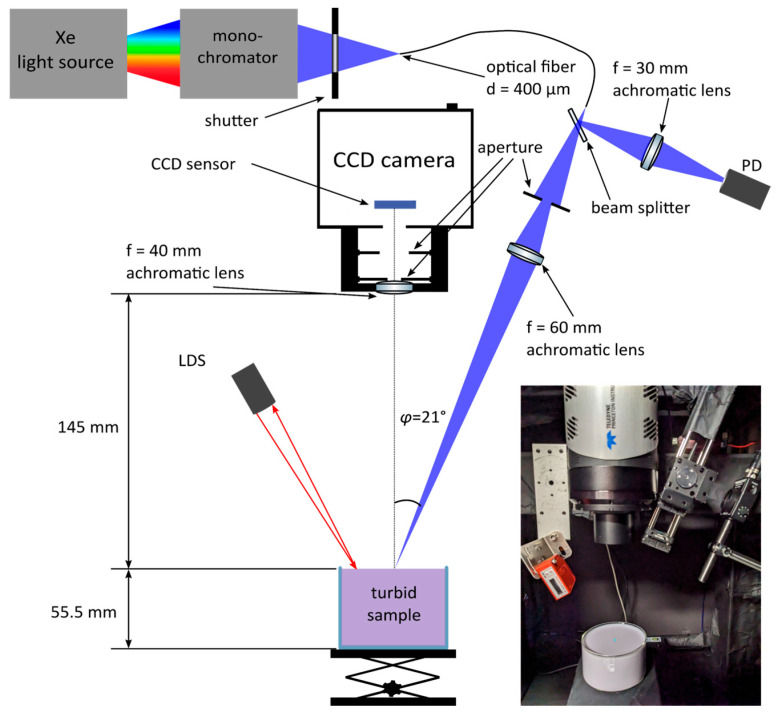
A schematic of the SRR imaging system, where PD and LDS represent a photodiode and a laser displacement sensor, respectively. A photograph is shown of the experimental system with the sample B.3 and the light source at 530 nm.

The SRR imaging system, shown in Figure 1, consisted of a Xenon light source and controller (SLS401, Thorlabs, Inc., Newton, NJ, USA), with output directed into a monochromator (Omni-λ 150, Zolix Instrument Co., Ltd., Beijing, China). The monochromatic output passed through a homemade shutter controlled by the data acquisition software described in Section 2.3. Incident light was supplied by an optical fiber with an output diameter of 0.4 mm at an angle of 21° from the plane normal to the sample. Immediately after the fiber output, the beam was passed through a beam splitter, directing about 8% of the incident light intensity to a photodiode (PDA100A2, Thorlabs, Inc., Newton, NJ, USA) to collect a reference signal of the incident light for normalization of all the acquired images. The remaining portion of light was focused to a radius *ρ*_r_ of 0.27 mm at the sample surface, using a 60 mm focal length achromatic lens (Linos, Excelitas Technologies Corp., Pittsburgh, PA, USA). The imaging system consisted of a 16-bit CCD camera (PIXIS 1024, Teledyne Princeton Instruments, Trenton, NJ, USA) to acquire a 1024 × 1024 pixel image while the sensor was cooled to −70 °C. A single achromatic lens of a 40 mm focal length (Linos, Excelitas Technologies Corp., Pittsburgh, PA, USA) was positioned prior to the camera via a C-mount adapter (SM2A6, Thorlabs, Inc., Newton, NJ, USA) together with a stainless-steel aperture (PKIT4K, Thorlabs, Inc., Newton, NJ, USA) of a diameter of 2.5 mm to minimize artifacts in the resulting images. During testing with a diffusive reflecting standard of 4000 grit sandpaper (Technodisc SiC, Kulzer GmbH, Hanau, Germany), it was observed that the stainless-steel aperture resulted in artifacts in long-integration time images, resolved by spray-painting the apertures matte black (DUPLI-COLOR 385872, European Aerosols, GmbH, Haßmersheim, Germany). To further improve image quality, a second, larger aperture of the same kind with a 5 mm diameter was placed behind the 2.5 mm aperture, minimizing the detection of internally scattered light by the objective components.

Samples were positioned on a stage with an adjustable height to ensure an identical beam incident on samples with differing thicknesses. To further ensure consistency in the height of the sample across multiple measurements and sample types, a laser displacement sensor (ODS9L2.8/LFH-100-M12, Leuze electronic GmbH, Owen, Germany) was used during alignment and subsequently switched off to avoid stray light from its LEDs and the display window. The voltage output of the laser displacement sensor during sample stage adjustment was noted and saved for measurements of unknown samples to ensure consistency of the pixel scale across multiple measurements.

### 2.3. SRR Imaging System: Software

A comprehensive GUI was written in the language of Python (version 3.8.10) for automated acquisition of reflectance images. A window showed the log-scaled image that was most recently acquired during an acquisition procedure, along with the radially binned plot for reference for the user. Measurement parameters were available to the user in a settings menu for adjusting integration time, number of repetitions, and spectral range. The laser displacement sensor was controlled by this software, with a real-time display as the sample stage was adjusted. Also displayed in real time was the incident light monitoring photodiode voltage, important for use as an indicator of light source stability during the warm-up period after powering on the system. The external shutter, used for obtaining dark images during experimentation, along with the photodiode, was controlled via an ADC (RedLab 1608FS, Meilhaus Electronic, Alling, Germany) interfaced with the control software. Communication with the camera and monochromator was performed via USB and serial port, respectively, each using the Python control software. Many functions were available to the user via button presses, such as an algorithm for the determination of the optimal integration time, which results in a maximum pixel intensity specified by the user. In our case, with a 16-bit depth, we typically optimized the integration time such that a maximum pixel intensity of 30,000 counts was obtained to avoid pixel saturation and blooming of the CCD sensor. An algorithm for pixel scale determination was also available to the user, wherein a reference object of known dimensions was imaged and the pixel scale calculated for later use in radial binning calculations.

During image acquisition, the TTL output from the camera was used to trigger a polling thread in the acquisition software, which sampled the photodiode voltage at a frequency of 1200 Hz and acquired an average over the duration of image acquisition. This voltage, along with the integration time for a given measurement, was saved in a .txt file for later use in normalization of reflectance images. In the case of long integration times in which the light source intensity may drift over longer time scales, we also considered algorithms for normalizing by a weighted average of the time-dependent intensity; however, in all results presented in this work, only the mean photodiode voltage was used for normalization.

### 2.4. Beam Characterization

**Figure 2:**
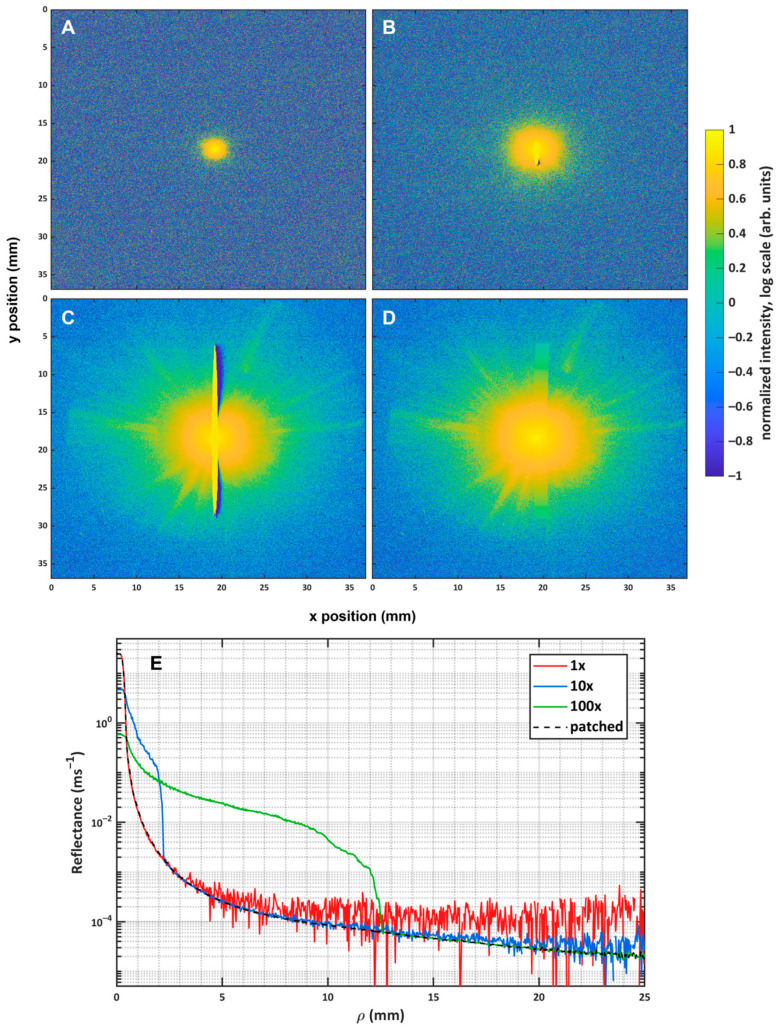
A beam characterization using the sandpaper reference. (**A**–**C**) A display of the 1×, 10×, and 100× dark-corrected images, with each obtained using the sandpaper reference. (**D**) Images resulting from patching all three 1×, 10×, and 100× integration time images. Each image has been normalized and plotted on a log scale. Thus, the color scale should not be used for quantitative comparison between images. (**E**) A comparison between the absolute radial binned reflectance obtained from the 1×, 10×, and 100× images, compared to the curve resulting from the patched image of (**D**).

We measured our incident beam profile at wavelengths of 500 to 800 nm in 20 nm steps using imaging 4000 grit sandpaper (Technodisc SiC, Kulzer GmbH, Hanau, Germany). Images acquired at low integration time characterized the beam profile at small radial distances *ρ* from the source but lacked information at large *ρ*, as noise dominated the measured pixel intensity in this regime. To acquire information regarding the system response at large *ρ*, we acquired two more images with integration times at a factor of 10 and 100 larger than that used in the first image. These three images are termed “1×”, “10×”, and “100×”, taken in succession for a given wavelength. An example of these images obtained at 660 nm is shown in Figure 2A–C.

Each image carries unique information regarding the beam and system response at differing *ρ*, with each displaying a reflectance of a suitable SNR at quite different regions of *ρ*. A stitched composite of the three individual images was used as the measured profile; however, during 10× and 100× imaging, the CCD sensor exhibited significant blooming due to saturation. An algorithm was developed such that the bloomed pixels of the 10× and 100× were neglected, the radial average was taken of the remaining pixels, and those neglected bloomed pixels were back-filled with the radial average, according to their *ρ* position. The bloom-corrected images were then stitched together to form a two-dimensional image representative of the incident beam profile and instrument response at small and large *ρ*. Figure 2D,E show the patched image and the resulting radially binned reflectance profile, respectively. The curve arising from the patched image follows each of the 1×, 10×, and 100× in succession until the SNR of each is deemed to be too low. One can observe that the use of the 100× allows for increased dynamic range by ensuring that the reflectance at large *ρ* can be measured above the background, useful for capturing the response of the imaging system in long integration time scenarios. The patched beam profile image was measured for each considered wavelength and reserved for use to describe the beam profile applied in MC simulations for the inverse recovery of SRR optical coefficients. We note that in addition to the beam profile, the image in Figure 2D contains information regarding the PSF of the system, which serves to improve the agreement between experimental and simulated reflectance.

### 2.5. System Calibration and Scaling Coefficient Calculation

A series of calibrations was performed prior to the use of the SRR imaging system for measurements of the sphere–suspension samples. A flatfield correction was applied by imaging with an in-house developed diffuse light box with three plexiglass diffusive sheets of a size of 250 mm × 250 mm × 5 mm illuminated from below by LEDs. The surface of the uppermost plexiglass sheet was aligned to the imaging plane such that the incident light beam was located at the origin used in Section 2.4. Illumination was provided only by the light box, and images were obtained with the light box in five different locations in the lateral plane to account for inhomogeneities in its output light distribution. It was determined that there was no more than a 5% difference in imaged intensity between the origin pixel and those near the image borders. A two-dimensional flatfield correction image was obtained and applied to all images in the post-processing stage.

To enable the recovery of absolute optical properties by an SRR system, one must account for the systematic differences between the experimental and digital systems. Our MC simulations were normalized to the incident number of simulated photons, although experimental image pixel response is dependent on factors that cannot be easily modeled in MC simulations, such as light source output intensity, CCD sensor sensitivity, and integration time. A direct measurement of incident light power, which can be correlated to pixel intensity, would be ideal, as it allows for reflectance to be measured as a ratio to be compared directly to that obtained by an MC digital twin. This approach, however, would require one to measure a spectrally resolved conversion factor at many different integration times, which may be used during an arbitrary experimental measurement or to assume a linear relationship between conversion factor and integration time. In our system, with measurements from 500 to 800 nm, integration times varied by up to two orders of magnitude across the eight sphere–suspension samples. In principle, one could predetermine integration times to be used at given spectral regions for each sample and measure a conversion factor at each. However, such an approach would greatly increase the measurement demand and significantly reduce the flexibility of the system regarding the variety of samples that could be measured with a good SNR and the spectral range of those measurements.

Our approach to enable absolute fittings was to use a well-characterized reference sample to acquire a scaling coefficient *k* between experimental and MC-obtained reflectance. The MC code used to obtain all SRR curves featured a homogenous volume of a specified thickness *d*_SRR_ equal to that of the experimental sample, with a two-dimensional detector confined to the sample surface. Incident light was injected into the sample with the two-dimensional beam profile measured in Section 2.4, with an angle relative to the plane normal to the sample surface calculated by Snell’s law between the air and water interface. Single photons were tracked as governed by the optical properties of the medium, and if the photon refracted through the sample–air interface, its location was recorded as the detection location on the virtual detector. Thus, a solid angle of detection was not used. The scaling coefficient *k* was calculated as

(1) $$k(\lambda ,\rho )=\frac{R_{MC}(\lambda ,\rho )}{R_{exp}(\lambda ,\rho )},$$

where *R*_MC_ is the radially binned reflectance arising from MC-obtained two-dimensional simulations, using optical properties measured by integrating sphere, and *R*_exp_ is that arising from the experimental SRR image. Thus, the measurement of *k* calibrates the SRR system against the well-validated, in-house integrating sphere system described in Section 2.1. In the scope of this work, the scaling coefficient *k* possessed a unit of [V∙ms], arising from the incident light monitoring photodiode and integration time normalizations, as each reflectance term possesses a [mm^−2^] unit, leaving only a dependence on experimental system parameters. The average *k* over a range of *ρ*, $\bar{k}(\lambda )$ is given as

(2) $$\bar{k}(\lambda )=\frac{1}{P}\sum_{\rho =\rho_{0}}^{\rho =\rho_{m}}\frac{R_{MC}(\lambda ,\rho )}{R_{exp}(\lambda ,\rho )},$$

where the bounding positions of *ρ* are given by *ρ*_0_ and *ρ*_m_, with *ρ*_0_ = *ρ*_r_ + *dρ*, where *dρ* is the bin width in the radial binning algorithm, held constant in this work at the side length of a single pixel, 0.0361 mm. The upper bound *ρ*_m_ is the radial distance at which the condition *R*_exp_(*ρ*_0_)/*R*_exp_(*ρ*_m_) = 10^3^ is satisfied. The summation is performed such that *P* discrete values of reflectance contribute to the calculation of $\bar{k}$, including those at the *ρ* bounds, with such bounds chosen empirically according to the elevated noise in radially binned reflectance after three orders of magnitude attenuation. All experimentally obtained images, before use in the LM fitting algorithm, were scaled by multiplying each pixel of the two-dimensional image with $\bar{k}$ from the reference sample. Radially binned reflectance arising from the scaled experimental two-dimensional image is written as *R*_exp,sc_ = $\bar{k}$ ∙ *R*_exp_. For all fittings presented in this work, the sample A.2 was chosen to serve as the reference standard from which $\bar{k}$ was calculated. A roughly linear increase in $\bar{k}(\lambda )$ was observed in the measurements over the spectral range of 500 to 800 nm. All measurements in this work were performed with the absolute measured values at each wavelength, but for reference, a linear fit gave a relation of $\bar{k}$(*λ*) = 0.069 × 10^−6^∙*λ* V∙ms + 2.182 × 10^−6^ V∙ms, with wavelengths in the unit of nm.

### 2.6. Recovery of Optical Coefficients by SRR Imaging System

Prior to SRR measurements, the mixing procedure, as described in Section 2.1, for the integrating sphere reference measurements was applied to the stock sphere–suspension solutions. A glass beaker of 60 mm diameter and *d*_SRR_ = 55.5 mm was filled with the mixed sample. Aided by the laser displacement sensor, the height of the sample stage was adjusted until the beam was centered at the identical pixel at which the beam profile was measured with the sandpaper reference described in Section 2.4, ensuring a consistent working distance and therefore pixel scale, minimizing the *ρ*–bin mismatch between *R*_MC_ and *R*_exp,sc_. All eight samples were measured using the SRR experimental system, with four repetitions for each over the range of 500 to 800 nm in 20 nm steps in a cyclic fashion, such that an entire wavelength cycle was completed before beginning the next repetition. As a full wavelength cycle required about 20 min to complete, the cyclic measurement procedure was chosen to reveal any time-sensitivity in sphere–suspension optical properties due to sedimentation or agglomeration.

An LM inversion algorithm was developed, which minimized the cost function *χ*^2^, which, at a given wavelength and during a given iteration of the LM algorithm, was calculated as

(3) $$\chi^{2}=\frac{1}{P}\sum_{\rho =\rho_{0}}^{\rho =\rho_{m}}{\left[\frac{log\left(R_{MC}(\rho )\right)-log\left(R_{exp,sc}\left(\rho \right)\right)}{\sigma \left(\rho \right)}\right]}^{2},$$

where *σ* is the *ρ*-resolved weighting function. In the scope of this work, *σ* = 1 was chosen for all fittings. To build the Jacobian used in the LM algorithm, partial derivatives of *R*_MC_ with respect to a given optimization parameter *p* must be obtained. In the context of our radially binned reflectance approach, these were written as

(4) $$\frac{\partial R_{MC}}{\partial p}=\frac{R_{MC}(p^{\prime })-R_{MC}(p)}{p^{\prime }-p},$$

where *p*′ is a perturbed value of *p*, thereby invoking the finite difference method. As has been demonstrated in a recent work from our group [22], we can use perturbation Monte Carlo methods to obtain computed simulations with small perturbations to optical properties rather than repeating the entire stochastic simulations [39], reducing computational cost and allowing us to rewrite Equation (4) as

(5) $$\frac{\partial R_{MC}}{\partial p}=\frac{S_{p}(R_{MC}(p))-R_{MC}(p)}{p\delta_{p}},$$

where *δ*_p_ is the size of the perturbation and *S*_p_ is a so-called scaling function, described in detail elsewhere [22]. Each derivative, with respect to both *µ_a_* and *µ_s_*′, is calculated, which allows for the construction of the Gauss–Newton equation and the optimization of *χ*^2^.

For fittings of all the wavelengths of all the samples, the initial guess in the LM search was constant at 0.01 and 1.0 mm^−1^ in *µ_a_* and *µ_s_*′, respectively. As in the case of integrating sphere measurements, *g* was held constant at 0.8 for all fittings using an HG-approximated phase function, and *n*(*λ*) of the medium was equal to that of water. In MC simulations to obtain reference SRR curves, there were multiple choices for integration of the measured beam profile in Section 2.4. Initially, our approach involved performing a convolution of a pencil–beam obtained MC two-dimensional image, with an experimentally measured beam profile image of the same dimensions. However, we observed a bottleneck in computation time in the LM algorithm, where the convolution of two 1024 × 1024 pixel images during each LM iteration resulted in a significant slowdown. Rather, the two-dimensional beam profile images were integrated directly in the MC simulations, where the spatial distribution of the incident beam locations was informed by a random distribution weighted by the pixel intensity of the reference image. We verified that an identical image was obtained with either method under the case of identical optical properties, but observed that the convolution-based method was about a factor of two slower than the integrated beam profile method for a complete 16 wavelength optimization, with no improvement in fitting quality compared to integrating sphere obtained optical properties. Therefore, all fittings presented in this work were obtained using the integrated beam profile method. The LM optimization had a duration of about 4 to 7 min for a given optimization routine. This time can be reduced in future work by decreasing the stringency of cutoff conditions and modifying other LM parameters such that the algorithm more rapidly progresses towards the solution. The results presented in this work were obtained by adjusting LM fitting parameters, such as the damping parameter, to prioritize accuracy and repeatability at the expense of time. We note that in the future, a neural network could be implemented, which would likely shorten inverse fittings to the seconds or even millisecond scale.

## 3. Results

**Figure 3:**
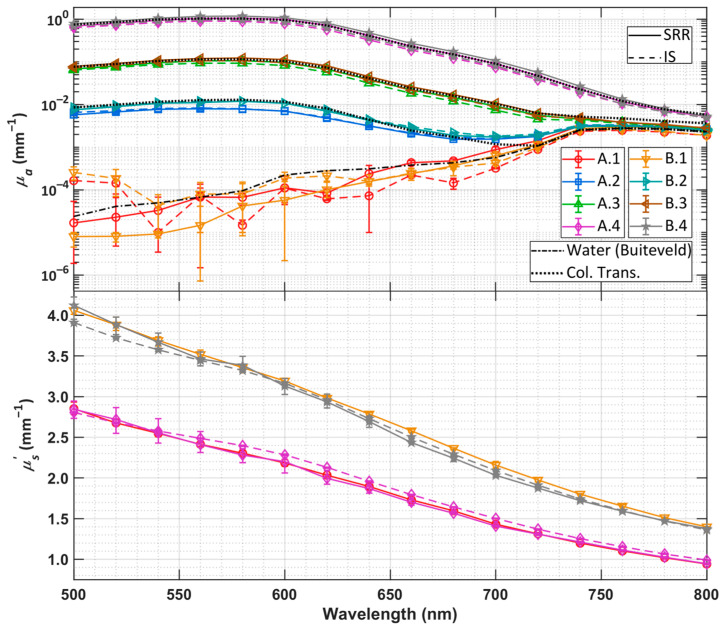
The fitted optical coefficients *µ_a_* and *µ_s_*′ from 500 to 800 nm in 20 nm steps. Only a selection of *µ_s_*′ fittings are shown to ensure readability of the plotted data. The solid and dashed lines denote the integrating sphere (IS) reference and SRR measurements, respectively. The error bars determined by the minimum and maximum of a given fitting across multiple measurements of the same sample. In most cases, the error bars are too small to be seen by eye. Reference measurements of *µ_a_* obtained by collimated transmission (Col. Trans.) are shown by the black dotted lines for each sample with added ink, and that of water is also provided, which was compiled from [40].

LM fittings obtained by the SRR imaging system are compared to those obtained by integrating sphere reference and collimated transmission systems and are shown in Figure 3.

The fittings of all samples appear to agree well with the integrating sphere and collimated transmission reference measurements. Spectral trends in IS reference *µ_a_* and *µ_s_*′ are clearly preserved, and absolute values of each are well matched. In most cases, the error bars appear quite small, suggesting that the SRR imaging system provides fittings with good repeatability and that the sphere suspensions did not exhibit deleterious sedimentation behavior. For samples A.1 and B.1, which had no added ink, the fitted *µ_a_* displays very large uncertainty, which is expected, as the absorption in such a sample is due only to that of water, which is quite small in the lower range of the considered wavelengths. Nonetheless, it appears that the mean fitted absorption in these cases closely follows the reference values of water absorption published in the literature [40], especially in the spectral range of 640 nm and longer. The absorption of each sample with added ink (A.2-4 & B.2-4) shows excellent agreement with the collimated transmittance-obtained values. Differences in the longer wavelengths can be explained by the very low attenuation of the samples in this spectral range, demonstrating that SRR and IS methods appear to be preferable over collimated transmission for samples of very low absorption. Differences in fitted and reference *µ_a_* seem to increase as the concentration of ink increases, as suggested by the A.4 and B.4 samples differing from the IS reference measurements by the largest margin of any samples measured in this work. While still relatively small, *µ_s_*′ variance seems to be the largest in highly absorbing samples in which *ρ*_m_ is quite small at no more than two millimeters. Across all samples and all wavelengths, *µ_s_*′ fittings are obtained with no more than 6% relative difference to the IS reference measurements.

**Figure 4:**
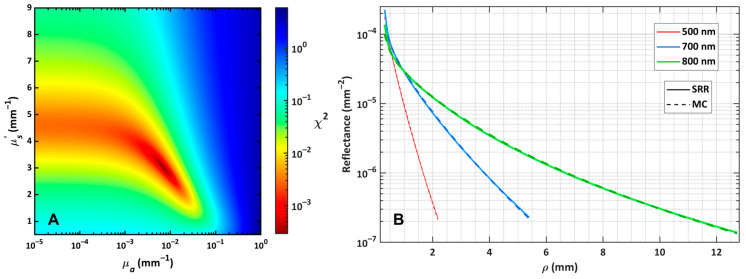
(**A**) The distribution of *χ*^2^, as calculated by Equation (3) over the *µ_a_*-*µ_s_*′ dimensions, demonstrates a well-posed search space observed for the sample B.2 at 620 nm. (**B**) A comparison of the radially-binned reflectance of the experimental image and the MC-simulated image with the best fitting optical properties *µ_a_* and *µ_s_*′ for the three wavelengths of the A.4 sphere–suspension sample.

An example of the two-dimensional *µ_a_*-*µ_s_*′ search space over which *χ*^2^ was minimized is shown in Figure 4A for the B.2 sample at 620 nm, demonstrating that the LM-search spaces were well-behaved and did not exhibit local minima.

A selection of *R*_exp,sc_, with comparison to the corresponding *R*_MC_ calculated with the fitted values of *µ_a_* and *µ_s_*′, is shown in Figure 4B for the A.4 sample at three wavelengths. Each curve is plotted between the bounds of *ρ*_0_ and *ρ*_m_, which vary for each wavelength. The reflectance curves display a strong sensitivity to the optical properties, with samples having high ink concentration attenuating very quickly. Despite only about 1.5 mm of *R*_exp,sc_ contributing to the LM fitting algorithm, the optical properties of the SRR system agree well with those obtained by integrating both sphere and collimated transmission.

## 4. Discussion

We have demonstrated that our SRR imaging system is able to accurately recover the optical coefficients *µ_a_* and *µ_s_*′ in absorbing turbid samples over a broad spectral range, with good agreement with values obtained by integrating sphere and collimated transmission methods. Compared to previous systems, we have included additional hardware to improve repeatability of measurements, used sophisticated MC simulations with the unique beam profile of the experimental system to recover simulated reflectance, and determined absolute fittings of samples to a large range of absorption and scattering, as demonstrated in Figure 3 and Figure 4. The fitted scattering coefficient varied by a factor of about 4, and the absorption coefficient by about 4 orders of magnitude, covering most of the values measured for biological tissue in the visible and infrared wavelength ranges [41,42]. The LM search space in the *µ_a_*-*µ_s_*′ plane demonstrates that the inversion procedure is well-posed and that we expect to determine a global minimum value of *χ*^2^ without concern for local minima, as may be the case when applying a single-layer homogenous reference model for fitting reflectance arising from multiple layers [43,44]. We observe that the calculated *χ*^2^ solution for this sample of relatively low absorption is much more sensitive to changes in *µ_s_*′ than to changes in *µ_a_*, a typical behavior that is observed in samples of low absorption in the range of 10^−3^ mm^−1^ or smaller. In Figure 2, we observe, with comparison to the literature-reported measurements of the absorption coefficient of pure water, that the lower-bound of measurable *µ_a_* using the SRR experimental system appears to be lower in magnitude than that of the integrating sphere system, demonstrated by lower wavelength fittings of *µ_a_* near 500 nm for the samples A.1 and B.1. We attribute the improved sensitivity to low *µ_a_* by the SRR experimental system to the contributions of reflectance collected at large SDS, thereby sampling photons which have traveled a long path through the medium, which is not possible in an integrating sphere system. Thus, in comparison to integrating spheres in the context of optical metrology and characterization, SRR systems may be ideal for measurements of minimally absorbing samples.

We assert that the SRR system presented in this work has been validated as a useful method for accurate retrieval of optical coefficients of turbid media. The disagreements that we have shown to exist between the SRR and reference optical coefficients can be improved by addressing multiple factors in the experimental system and the fitting algorithms. Challenges experienced during the development of the SRR system are discussed below, along with their current outlook and an appeal to future work.

### 4.1. Determining the Point of Beam Incidence

The correct determination of the point of light incidence on the sample surface is extremely important to the accuracy of fitted optical properties, and this point must be consistent across measurements of the beam profile and those for all unknown samples. The beam center is taken as the origin during radial binning, and if its location is determined incorrectly, *R*_exp,sc_ and *R*_MC_ may possess different origins, resulting in a *ρ*-offset of radial profiles such that the cost function *χ*^2^ is calculated from reflectance at different distances from the origin, introducing significant error in the fitted coefficients. The laser displacement sensor discussed in Section 2.2 is useful for coarse alignment to bring the incident beam within about 10 pixels (~0.3 mm) of the intended single-pixel origin coordinate pair. However, in long-term time scales over many measurement events, due to vibrations and perturbations in the laboratory caused by footsteps and the opening and closing of doors, for example, small changes in the laser displacement sensor output for the same sample stage height have been observed, resulting in *R*_exp,sc_ and *R*_MC_ origin mismatch of a few pixels when relying only on the laser displacement sensor output. A center of mass (COM) algorithm was tested for automated calculation of the center point, but due to the oblique angle of incident light, the imaged beam profile is not symmetric, depending on the optical properties of the sample. Therefore, a COM calculation is typically unable to find the center of the incident beam correctly. Additionally, the precision of determining a true origin of an image is inhibited by the discretized nature of the pixel grid, resulting in a tolerance of one pixel, or about 0.0361 mm in the case of our system’s typical WD and pixel scale. Further, when imaging a diffusive medium, the image that one obtains is not exactly an image of the surface, but also includes light from subsurface scattering. Because a suitable automated method was not determined, fine alignment of an experimental sample to the exact pixel used during beam profile measurements was performed by the eye of the user.

Manual alignment resulted in the origin of the *R*_exp,sc_ and *R*_MC_ differing by no more than a single pixel in the *ρ*-dimension. We tested how a single-pixel mismatch would affect calculated *χ*^2^ and optical property fittings by manually shifting one of the reflectance curves relative to the other by a single pixel and refitting optical coefficients for all sixteen wavelengths. We observed a factor of ≤1–2% difference between the optical coefficient fittings of shifted and unshifted cases. However, we observed a *χ*^2^ of about a factor of 2 larger in the case of mismatched origins, suggesting that while the *ρ*-mismatched *R*_exp,sc_ and *R*_MC_ show significantly poorer agreement than with origin matching, fittings of *µ_a_* and *µ_s_*′ appear fairly robust against a single-pixel difference in the determination of the origin. Nonetheless, progress is underway to determine an algorithm for automated or computer-assisted methods for more robust operation in the case of unsupervised use or usage by non-experts. One idea is the detection of specular reflection at an angle of 360 − *φ* degrees through a pinhole (see Figure 1), filtering out the volume-reflected light.

### 4.2. Choice of Reference Phantom

In this work, the calculation of the scaling coefficient *k* is dependent on the choice of reference phantom used for comparison between MC and experimental curves. The choice of reference sample is determined by its homogeneity and the smoothness of its surface. The quality of *k* in terms of SRR fittings is also dependent on the quality of *µ_a_* and *µ_s_*′ fittings obtained by the reference method, in our case, the integrating sphere system. Thus, in general, the reference phantom should not possess excessively large or small optical properties that are not well handled by the integrating sphere. Intralipid^®^, an organic lipid emulsion, is commonly used as an optical standard for validation of optical systems [28,31,32,45,46,47] and was considered as a candidate reference standard. However, we have observed in the past that these emulsions tend to form a distinct surface layer visible to the eye [48], rendering the sample inhomogeneous and thus not matching our homogenous MC volume. Similarly, bovine milk has gained interest as an optical standard [25,49,50,51,52], but these samples tend to suffer from similar tendencies. When we constructed a sample of commercially obtained homogenized bovine milk, we saw a clear layer on the surface attributed to the local accumulation of fat cells. We also considered solid polydimethylsiloxane (PDMS) phantoms as the reference sample, but surface roughness and inhomogeneities were detrimental to their ability to provide a useful *k*.

Polymer microsphere suspensions were chosen as the reference sample to overcome the challenges discussed above. The water medium ensured a smooth surface that eliminated the measurement of scattered light due to surface roughness, resulting in excellent matching to the MC model. For a consideration of surface roughness in SRR, which was not included in this work, we point the reader to a previous publication [53]. The moderate concentration of ink resulted in a sample with absorption comfortably above the lower limit measurable by the integrating sphere, allowing a larger number of radial distances over which we could calculate $\bar{k}$ compared to that obtained from a sample with higher absorbance. However, polymer microspheres have a tendency to form agglomerations, preventing a monodisperse distribution in the sample volume not accounted for in an MC simulation. Thus, it was necessary for the concentration of scatterers not to be excessively high and to ensure proper mixing to prevent agglomeration of microspheres. If not properly mixed, one was able to see streaking and thus an inhomogeneous distribution of scatterers. The criteria of the choice of reference, while reasonable, were empirical, and in principle, the choice of such a reference is open to the researcher. Our choice is supported by the extensive evidence presented in this work, in which numerous samples of widely varying optical properties are fitted well by the LM algorithm and compare quite well to the reference integrating sphere-obtained optical properties; however, other sample types could theoretically be used to achieve similar results. Testing is underway with improved PDMS solid samples with smooth surfaces [54], which are ideal as they have a longer shelf-life than a sphere–suspension in water and would provide information about system status with the ability to perform measurements both in short-term and long-term time scales.

It can be seen in Figure 3 that the fitting of sample A.2 displays optical coefficients that still possess errors relative to the integrating sphere reference, despite the fact that $\bar{k}$ was calculated by the experimental images arising from this same sample. Because the fittings obtained for this sample differ from the integrating sphere reference, it can be concluded that there remain deficiencies in the use of a scalar correction factor $\bar{k}$. Because *k* exhibits *ρ* dependence, the correction of experimental images by $\bar{k}$ is the application of a DC shift, rather than a *ρ*-resolved correction, which considers more rigorously spatial differences in light detection between the digital and experimental system. We also tested the use of the unaveraged, *ρ*-resolved *k* by performing a bin-wise application of the scaling coefficient on experimental radially binned reflectance. This method required a reference sample in which a reflectance curve could be obtained with a good SNR over the entire range of *ρ* that may be used in the measurement of any future sample. Thus, a sample with large attenuation is not ideal due to the inapplicability of its obtained rho-resolved *k* for samples of low attenuation. Fittings with rho-resolved *k* were consistently less similar to the integrating sphere-obtained fittings than those obtained with $\bar{k}$. Future work could consider the reason for variation in *k* with *ρ* as a means to further improve the accuracy of SRR-obtained optical coefficients.

### 4.3. Lambertian Reflectance Standard

**Figure 5:**
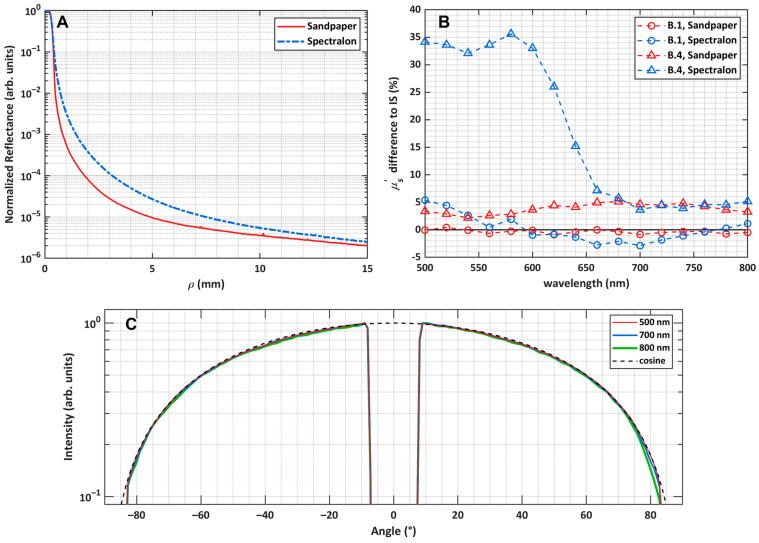
(**A**) A comparison of radially binned beam profiles of the experimental system measured by two standards. (**B**) The relative difference in the *µ_s_*′ fittings for two samples compared to those by integrating sphere (IS), depending on the choice of standard used for beam profile measurement. The black line is a reference for the viewer at 0%. (**C**) The goniometric measurement of reflected light by sandpaper at three wavelengths compared to a cosine distribution at perpendicular light incidence. Each curve is normalized against its maximum.

In the measurement of the beam profile in the fashion presented in Section 2.4, we required a diffusely reflecting surface from which scattered light is imaged by the CCD sensor and the beam profile is measured. Ideally, a standard possessing a Lambertian scattering profile would be used, such that we obtain the instrument response of our SRR system. At the moment, a fine-roughness sandpaper of low cost was used to obtain such profiles. As a comparison, a Spectralon^®^ 99% (Labsphere, Inc., North Sutton, NH, USA) reflectance standard, which the manufacturer states as having Lambertian reflectance, was used to obtain the beam profile for MC input in the same fashion as was used with the sandpaper. Radial binning was applied to the two-dimensional image obtained from the patching algorithm described in Section 2.4 and is shown in comparison to the sandpaper radial profile below in Figure 5A.

Figure 5A demonstrates that the radially binned beam profile arising from the sandpaper standard possesses a much steeper slope in the region immediately outside of the beam radius. That obtained from the Spectralon sample, however, features a broader profile, demonstrating that the small transmission inherent in the Spectralon sample results in this sample being inappropriate to measure an accurate beam profile of the experimental system due to volume scattering. The effect of a Spectralon-measured beam profile on fitted optical properties is seen in Figure 5B, using the B.1 and B.4 sphere–suspension samples. These particular samples were chosen because of the large range of attenuation over the considered spectral range. The fitted optical properties differ significantly across the two methods, with the severity of disagreement appearing to be related to the degree of light attenuation by the sample. At low wavelengths, where *µ_a_* is high, the deviation in fitted *µ_s_*′ relative to integrating sphere fittings is quite high in the case of a Spectralon standard. At large wavelengths, when *µ_a_* is smaller and similar between the B.1 and B.4 samples, it appears that the choice of reference is less impactful on the fitted *µ_s_*′. This may be explained by an overestimated beam profile, which arises with a Spectralon standard, therefore causing a compensatory increase in *µ_s_*′. One can consider the measured experimental reflectance curve as a combination of the contribution of the beam profile and the diffusely scattered light that has propagated through the sample and remitted at some *ρ* from the source. In the case of a sandpaper standard, the beam profile is confined roughly to the true region of the beam, and thus, the contribution of the beam profile to measured reflectance at large *ρ* is much smaller than at smaller *ρ*. In the case of a highly attenuating sample, such as B.4 at short wavelengths, the reflectance contributing to the fitting belongs to a low *ρ* in the range of 1 to 2 mm. In this region, it is thought that the broad Spectralon beam profile contributes to the signal significantly more than that arising from sandpaper, thus overestimating the MC-simulated reflectance, demonstrating that a surface scatterer must be used in such a measurement. Finally, as demonstrated by Figure 5C, despite the low cost of the sandpaper, the reflectance profile at three wavelengths over the considered range appears to be quite Lambertian, with good overlap with a cosine distribution across a wide angular range, suggesting that the sandpaper is a good choice of reflectance standard.

### 4.4. Future Work

The validation of our experimental SRR imaging system enables the consideration of more complex samples, such as inhomogeneous phantoms with optically distinct layers and human biological tissue. We plan next to investigate recent findings by our group regarding the systematic errors that arise when modeling multiply-layered scattering media as a single homogenous layer during inverse fitting to experimentally validate our computational results [43,44,55]. We also plan to apply the system for a direct measurement of the in vivo optical properties of human tissue, for example, in the volar forearm or palm of the hand. Prior to an in vivo measurement of human skin, certain considerations must be observed and are discussed below.

As human skin, unlike the samples considered in this work, is not smooth and exhibits surface curvature, we must consider the response of fitted optical properties to such factors. We can rely on previous works, which have considered the effect of surface roughness on measured spatially resolved reflectance [53,56]. We note that the reflectance in the region nearest to the incident beam, which is most sensitive to surface roughness and is already neglected from contributing to optical property fitting. For the moderately to highly absorbing samples considered in this work, only reflectance up to about 3 to 5 mm from beam incidence was fitted, and we would expect a similar range during an in vivo measurement of human skin tissue, depending on the considered wavelength. Over such small distances, it is likely that a small degree of curvature should not be detrimental to fitted optical properties. However, if needed, we can integrate a tetrahedron–mesh Monte Carlo approach, as described in [57], to more accurately simulate the curved surface that is measured. Furthermore, the results obtained in this work used an HG-approximated scattering phase function with a constant *g* of 0.8. We did not observe significantly different fitted optical properties when our choice of *g* was modified by ±10%. However, in future work with in vivo measurements of human skin, it may be useful to consider other scattering phase functions, such as Reynolds–McCormick or the double HG.

Due to human tissues’ tendency to fluoresce under the excitation range presented in this work, it may be necessary to consider the detection of diffuse reflectance and fluorescence separately. We could perform tests using long-pass filters to determine quantitatively the effect of fluorescence on the measured reflectance signal to assess whether the inclusion of filters during optical property measurements is needed, though it is expected that the fluorescence signal is quite small in comparison to the diffuse reflectance. A thorough description of the discussed factors and others, such as polarization, could be investigated and presented in future work.

## 5. Conclusions

We have presented extensive details regarding the construction and validation of an SRR imaging system, which exhibits excellent agreement between experimentally determined optical coefficients and reference measurements performed by integrating sphere and collimated transmission approaches. The system demonstrated efficacy in measurements of both weakly and strongly attenuating samples, as fitted absorption agreed well with reference measurements over multiple orders of magnitude. There remain opportunities for improving the system by addressing standing difficulties in alignment and calibration; however, we have presented a clear roadmap forward, which we expect to further improve the already well-agreeing fitted optical coefficients to an independent reference. We conclude this work with a validated SRR system that is ready for applications to measure the optical properties of multi-layered phantoms and in vivo human tissue.

## Figures and Tables

**Table 1:** Sample naming conventions and their corresponding scatterer and absorber mass concentrations in pure water.

Sample Name	Base Scatterer Suspension (%)	Ink Absorber (%)
A.1	3.0	0
A.2	3.0	0.005
A.3	3.0	0.05
A.4	3.0	0.5
B.1	4.5	0
B.2	4.5	0.005
B.3	4.5	0.05
B.4	4.5	0.5

## Data Availability

Data underlying the results presented in this paper are not publicly available at this time but may be obtained from the authors upon reasonable request.

## Abbreviations

The following abbreviations are used in this manuscript:

SRR	Spatially resolved reflectance
RTE	Radiative transfer equation
HG	Henyey–Greenstein
MC	Monte Carlo
LUT	Lookup table
LM	Levenberg–Marquardt
NN	Neural network
ROI	Region of interest
SDS	Source–detector separation
WD	Working distance
PSF	Poin–spread function
IS	Integrating sphere
CT	Collimated transmission
COM	Center of mass
PDMS	Polydimethylsiloxane
